# Herb pair of Astragali Radix-Descurainiae Semen attenuate heart failure through the myosin VI-Tom1 complex mediated autophagy

**DOI:** 10.3389/fcvm.2025.1599746

**Published:** 2025-07-04

**Authors:** Mengyue Wang, Songlin Ni, Tong Wang, Mo Sun, Qiaolan Wu, Xiaolin Wu, Guangying Lu, Peiwei Su, Zu Gao, Qian Chen

**Affiliations:** ^1^ College of Traditional Chinese Medicine, Shandong University of Traditional Chinese Medicine, Jinan, China; ^2^ College of Nursing, Shandong University of Traditional Chinese Medicine, Jinan, China; ^3^ Shandong Co-Innovation Center of Classic TCM Formula, Shandong University of Traditional Chinese Medicine, Jinan, China; ^4^ Medicine College, Shandong University of Traditional Chinese Medicine, Jinan, China

**Keywords:** Astragali Radix-Descurainiae Semen, heart failure, autophgy, myosin VI-Tom1, TCM

## Abstract

**Aim of the study:**

This study aims to explore the therapeutic effects of Astragali Radix-Descurainiae Semen (AR-DS) on heart failure and elucidate the mechanisms behind its efficacy.

**Materials and methods:**

A rat model of heart failure was established and treated with various dosages of AR-DS decoction. Cardiac function was assessed using echocardiography, and cardiac-related mass indices were calculated. Histopathological changes were observed through HE and Masson staining. Serum levels of BNP, NT-pro BNP, and ANP were measured to evaluate AR-DS's efficacy. Electron microscopy was employed to examine the ultrastructure of cardiomyocytes, and TUNEL staining was used to assess apoptosis. Expression levels of LC3, Beclin1, p62, Myosin VI (MYO6), and Target of Myb1 (Tom1) in myocardial tissue were analyzed using qRT-PCR and Western Blot. The expression of MYO6 and Tom1 in myocardial tissue was observed through multiple immunofluorescent stainings. Protein docking was used to assess the binding energy between MYO6 and Tom1. Molecular docking to detect the binding energy and binding site of the MYO6-Tom1 complex to the major components of AR-DS.

**Results:**

AR-DS effectively improved cardiac function and mitigated myocardial pathology in heart failure rats; it reduced serum levels of BNP, NT-pro BNP, and ANP, and suppressed cardiomyocyte apoptosis; AR-DS significantly downregulated the gene and protein expression of LC3 and Beclin1, upregulated p62, and reduced autophagy in myocardial tissue; AR-DS can effectively down-regulate the gene and protein expression of MYO6 and Tom1 in heart failure rat myocardium; protein docking results demonstrated the formation of a stable MYO6-Tom1 complex; lastly, the molecular docking results showed that the binding energies of the main components of AR-DS: Ononin, Astragaloside-IV, Rutin, Folic-acid, Daidzein, Isorhamnetin, Quercetin, Beta-Sitosterol, Kaempferol, and Formononetin can bind to the MYO6-Tom1 complex.

**Conclusions:**

AR-DS exerts a protective effect on myocardial tissue in heart failure rats by inhibiting myocardial autophagy, potentially through the modulation of the MYO6-Tom1 complex. This offers new insights into the clinical treatment of heart failure.

## Graphical Abstract

Graphical Abstract. (Created using Figdraw).

**Figure:**
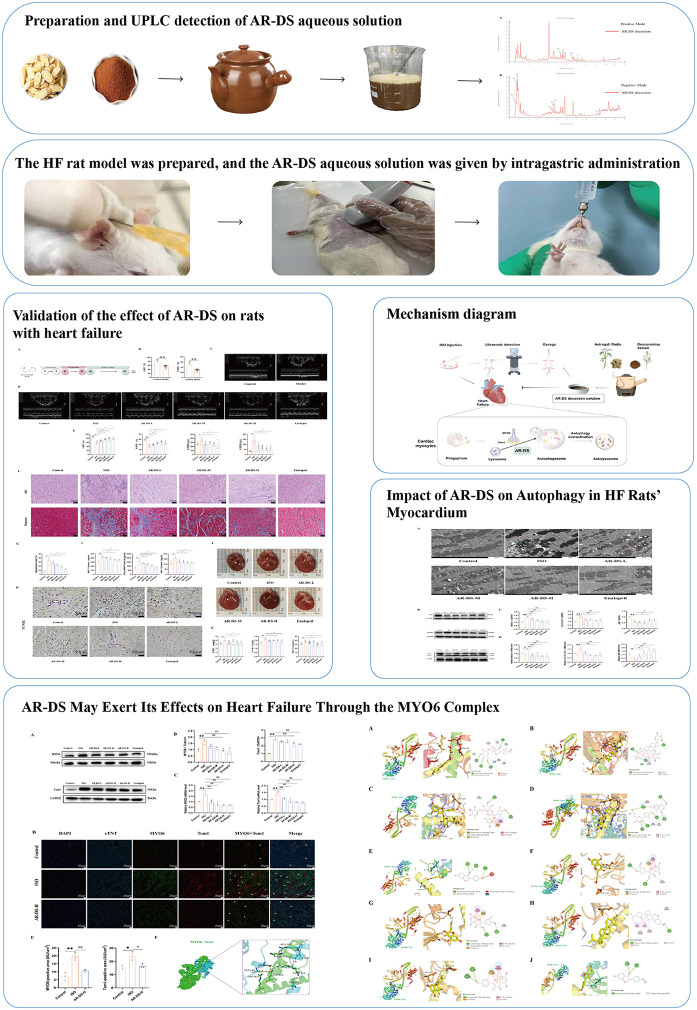

## 1 Introduction

Cardiovascular diseases rank among the top causes of global morbidity and mortality. Heart failure, as the ultimate outcome of various cardiovascular diseases, is a significant global public health issue (1, 2), accounting for 13% of the total global deaths (3). The prognosis for patients with heart failure is poor, with a 5-year survival rate of approximately 50% (4, 5). The etiology of heart failure is complex, and its pathogenesis remains unclear. Currently, common medications for heart failure primarily include diuretics, angiotensin-converting enzyme inhibitors, β-blockers, positive inotropes, and vasodilators, but their long-term use is associated with significant toxic side effects. For example, excessive diuresis can lead to dehydration or electrolyte disorders; angiotensin-converting enzyme inhibitors can cause postural hypotension, chest pain, stomach pain; beta-blockers can increase the occurrence of dizziness and bradycardia (3, 6). Thus, the development of safe and effective treatments for heart failure is still an unresolved issue.

Traditional Chinese Medicine (TCM) can potentially enhance outcomes in heart failure through various mechanisms, demonstrating promising application prospects (7). In TCM, heart failure is clinically categorized under syndromes such as cardiac pain, palpitations, and edema. These conditions are attributed to the dysfunction of internal organs, which results from the deficiency of Qi, blood, Yin, and Yang. This internal disharmony leads to a lack of necessary support for the heart. It results in impaired blood flow, leading to the congealment and blockage of blood, severely hindering circulation (8, 9). Heart Qi deficiency and internal retention of water are common syndromes observed in heart failure (10), and the treatment typically focuses on boosting Qi and facilitating diuresis (11). The combination of invigorating qi and diuretic drugs is a common combination of clinical heart failure treatment prescription. Astragali Radix (AR) has the power to strengthen Qi and promote diuresis while reducing swelling. Its active components, such as Astragalosides, can improve cardiac output, intervene in ventricular remodeling, and regulate neuroendocrine activities, making it a commonly used anti-heart failure herb (12). Descurainiae Semen (DS) can eliminate phlegm, relieve asthma, benefit the lungs, and reduce swelling. Its active component, descurainin, has effects similar to cardiac glycosides, which can alleviate heart failure by inhibiting the overactivation of the neuroendocrine system and enhancing myocardial contractility (13). AR and DS are tonic and diuretic drugs with boost Qi and facilitate diuresis. According to the research on the formula formation rule of heart failure based on the TCM inheritance assistance platform software, Astragali Radix-Descurainiae Semen (AR-DS) is the most frequent medicine pair that boosts Qi and facilitates diuresis (14, 15). Meanwhile, TCM formulations featuring AR-DS, such as the TCM Qiliqiangxin, have shown effective therapeutic effects on chronic heart failure by enhancing myocardial contractility, inhibiting the overactivation of the neuroendocrine system, and suppressing inflammatory responses (16). In addition, Liu Yan et al. (17) found that AR-DS is a multi-pathway and multi-targeted therapy for heart failure through network pharmacology. Yan Li et al. (18) found that AR-DS exerts anti-heart failure effects by affecting mitochondrial dynamics. The multi-target and multi-mechanism approach of traditional Chinese herbs in treating heart failure represents a highlight in heart failure prevention and treatment and poses a challenge in elucidating the pathways of herbal action. Thus, further exploration into the mechanism of AR-DS in treating heart failure is needed.

Autophagy is a well-preserved lysosomal process responsible for degrading proteins and organelles within cells, playing a crucial role in maintaining cardiovascular homeostasis (19). Under persistent adverse stress, excessive activation of autophagy not only exacerbates cardiomyocyte apoptosis and necrosis (20) but also leads to excessive deposition of myocardial collagen fibers, disproportion, disordered arrangement, and even ventricular remodeling (21), worsening cardiac function. Therefore, inhibiting overactive autophagy is an effective strategy to maintain the structural and functional stability of cardiomyocytes and prevent cardiac pathologies. The transport process of autophagy depends on the cytoskeleton (22), which is a spatial network structure in cells composed of fibrillar proteins, including microfilaments, microtubules, and intermediate filaments, among which microfilaments serve as the kinetic foundation for the occurrence and development of autophagy (23). Myosin, a molecular motor that relies on microfilaments, regulates multiple stages of autophagy (22). Myosin VI (MYO6), a type of cardiac myosin (24), is the only myosin motor in eukaryotic cells that moves along the surface of microfilaments towards the negative end, and its unique directionality allows it to control the maturation of autophagosomes and their fusion with lysosomes during the final stages of autophagy, participating in the formation of autophagosomes (25–28). Studies have confirmed that the Target of Myb1 (Tom1) is a binding partner of MYO6 on endosomes, and disruptions in the MYO6-Tom1 interaction can block the fusion of autophagosomes with lysosomes, inhibiting the development of autophagy (24, 29). Therefore, this unique cardiac myosin, MYO6, by inhibiting overactive autophagy and maintaining the homeostasis of cardiomyocytes, is a key target for the prevention and treatment of cardiac pathologies.

Therefore, we intervened in a rat heart failure model by replicating a rat model of heart failure and administering different concentrations of AR-DS decoction to evaluate the effects of AR-DS on intervening heart function in rats with heart failure through cardiac function and cardiac pathology alterations, and to validate the therapeutic efficacy of AR-DS in heart failure. The effects of AR-DS on cardiac autophagy status in heart failure rats through the MYO6-Tom1 complex were further explored to elucidate the mechanism of its action.

## 2 Materials and methods

### 2.1 Preparation and phytoconstituent analysis of AR-DS

Preparation of AR-DS decoction: Drugs: AR is the dry root of the leguminous plant *Astragalus membranaceus* (Fisch.) Beg. var. *mongholicus* (Beg.) Hsiao and *Astragalus membranaceus* (Fisch) Bge., are being used as a medicinal and edible resource (30). DS is the dried and mature seed of *Descurainia sophia* (L.) Webb. ex Prantl, known as “South draba seed”. AR and DS were authenticated by Prof. Fang Zhang of Shandong University of Traditional Chinese Medicine and conformed to the Pharmacopoeia of the People's Republic of China (2025 Edition).

AR and DS were selected from local herbs and decocted according to the highest adult clinical dose (30 g·70 kg^−1^ for AR, 10 g·70 kg^−1^ for DS) recorded in the 2025 edition of Chinese Pharmacopoeia. A predetermined amount of the drug was placed in a casserole dish with ten times the volume of distilled water and decocted for one hour. The decoction was then filtered and repeated three times, and then three times the filtrate was combined and concentrated at a constant temperature of 55℃ using a rotary evaporator. According to the equivalent dose coefficient discounting method, the equivalent dose for rats was calculated as 6.3 times that for adults according to the unit body weight, and the dose in the AR-DS decoction was concentrated to the concentration of the raw drug of 0.36 g·ml^−1^, and the two dose groups, high and low, were concentrated to 0.72 g·ml^−1^ and 0.18 g·ml^−1^ using the same method, and then refrigerated at 4℃ for use.

Sample extraction of decoction (31): Add 400 µl of methanol solution, vortex to obtain the supernatant, and filter it through a 0.22 μm membrane for LC-MS detection. Chromatographic conditions: Employed a Thermo Vanquish (Thermo Fisher Scientific, USA) ultra-high-performance liquid chromatography system with an ACQUITY UPLC® HSS T3 column (2.1 × 100 mm, 1.8 µm) (Waters, Milford, MA, USA) at a flow rate of 0.3 ml/min and a column temperature of 40 °C. 2103;. The injection volume was 2 µl. In positive and negative ionization modes, the mobile phase consisted of 0.1% formic acid in acetonitrile (B2) and 0.1% formic acid in water (A2), with a gradient program as follows: 0–1 min, 8% B2; 1–8 min, 8%–98% B2; 8–10 min, 98% B2; 10–10.1 min, 98%–8% B2; 10.1–12 min, 8% B2 (32, 33).

Mass Spectrometry Conditions: Utilizing the Thermo Orbitrap Exploris 120 mass spectrometer (Thermo Fisher Scientific, USA) equipped with an electrospray ionization (ESI) source, data were collected in both positive and negative ion modes. The spray voltage was set at 3.50 kV for positive ions and −2.50 kV for negative ions. A full-scan MS was performed at a resolution of 60,000 over a scan range of m/z 100–1,000. Secondary pyrolysis was carried out using High-energy collisional dissociation (34).

### 2.2 Animals and interventions

One hundred SPF-grade SD male rats, aged 8 weeks and weighing 200 ± 20 g, were procured from Beijing Huafukang Bioscience Co., Ltd., with license number SCXK (Beijing) 2019-0008. The rats were housed in an SPF-grade barrier facility with a temperature of (23 ± 2) ℃, humidity of 60% ± 10%, and a 12 h/12 h light/dark cycle. Animals had free access to food and water. All rats were acclimatized for one week before the experiment began. Animal experiments were conducted by the National Institutes of Health guidelines for the care and use of laboratory animals. All animal experiment protocols were approved by the Animal Ethics Committee of the Shandong University of TCM (Ethics Review Approval Number: SDUTCM20230621001).

Model Preparation: Ten rats were randomly selected to serve as the control group (Control), while the remaining rats were used to establish a heart failure model. The model group received subcutaneous injections of isoprenaline hydrochloride solution (3 mg·kg^−1^) (35–37). Isoprenaline hydrochloride (I5627) was obtained from Sigma-Aldrich (St. Louis, MO, USA). The Control group received equivalent doses of saline, with injections administered daily for a total of 10 days. On the 11th day, echocardiography was performed to assess heart function. Rats with a left ventricular ejection fraction (LVEF) of less than 60% and a left ventricular fractional shortening rate (LVFS) of less than 35% (38–41) were considered successful replications of the heart failure model. The 44 rats with successfully replicated models were randomly divided into five groups: the isoproterenol model group (ISO), the AR-DS low-, medium-, and high-dose groups (astragali radix - descurainiae semen low/medium/high-dose group, AR-DS-L/AR-DS-M/AR-DS-H), and the enalapril group. All groups were intervened with a 10 ml·kg-1 gavage volume on day 18. The three AR-DS administration groups were gavaged with the corresponding aqueous decoction. The enalapril group was gavaged with a suspension of 0.15 mg·ml^−1^ (equivalent to the clinical dose). Enalapril maleate tablets (Lot no: 210251) were sourced from Yangtze River Pharmaceutical Group, Jiangsu, China. The Control and ISO groups received equal volumes of saline gavage. All groups were gavaged continuously for 14 days, during which they were routinely raised. As shown in Figure 2A.

### 2.3 Evaluation of cardiac function by ultrasound

Rats were anesthetized with 3% isoflurane via inhalation and placed supine on a rat board, with the chest area prepared. An appropriate amount of coupling gel was applied to the skin surface, and a B-mode ultrasound probe was used to conduct cardiac ultrasonography. Once clear parasternal long-axis images were obtained, the M-mode ultrasound measurement line was aligned perpendicularly to the posterior wall of the left ventricle at its longest point to capture M-mode ultrasound images. Echocardiographic indices were obtained using ultrasound measurement tools, averaging each measurement over three consecutive cardiac cycles. All ultrasound examinations were conducted by a professional ultrasonographer.

### 2.4 Collection of animal tissue samples and assessment of relevant indicators

The rats were weighed and anesthetized with 3% isoflurane. After the blood was taken from the abdominal aorta, placed in the vessel at room temperature for 30 min, and centrifuged at 3,000 rpm·min^−1^ at 4°C for 10 min, the supernatant was taken and assayed for b-type natriuretic peptide (BNP), n-terminal pro b-type natriuretic peptide (NT-pro BNP), and atrial natriuretic peptide (ANP) according to the instructions of the ELISA kit (E-EL-R0017c, E-EL-R3023, E-EL-R0126c, Elabscience Biotechnology, Wuhan, China). The residual blood was cleaned with normal saline and dried with dust-free paper. The weight of the heart was measured by an electronic balance. The left atrium was cut along the coronary groove of the heart, the right ventricle was removed along the interventricular groove, and the left ventricular mass was measured. Remove the tibia and measure the tibia length. Heart index (heart mass/body mass), left ventricular mass index (left ventricular mass/body mass), and heart-tibial ratio (heart mass/tibial length) were calculated.

### 2.5 Staining

HE Staining and Masson Staining: The rat heart was quickly fixed in a 4% paraformaldehyde sampling bottle and was routinely dehydrated, embedded, and sliced, followed by HE staining and Masson staining.

TUNEL Staining: According to the kit (11684817910, ROCHE, Switzerland) instructions, Cardiac tissue sections were dewaxed and hydrated, followed by incubation with Proteinase K (20 μg·ml^−1^ dissolved in Tris/HCl, pH 7.4 8.0) at room temperature for 1,530 min and at 37°C for 15 min. After rinsing twice with PBS and drying the area around the sample, 50 μl of TUNEL reaction mixture was added and incubated at 37°C in a humidified chamber for 60 min. After washing three times with PBS, the samples were ready for analysis under a fluorescence microscope. The area around the sample was dried again, and 50 μl of converter was added before incubation in a humidified chamber at 37°C for 30 min. After three PBS washes, 50∼100 μl of DAB substrate solution was added and incubated at room temperature for 10 min, followed by three more PBS washes. Hematoxylin was used to counterstain the nuclei, after which the slides were mounted and analyzed under a light microscope.

Immunohistochemical Fluorescent Staining: Paraffin sections of rat hearts were routinely deparaffinized, and then the sections were placed in citric acid solution for antigen retrieval. Goat serum was applied to block the sections for 30 min. After removing the blocking solution, MYO6 primary antibody (1:200, 26778-1-AP, Proteintech, Wuhan, China) was applied and incubated at room temperature for 1 h, followed by washing with TBST. Secondary antibody was added and incubated for 10 min, followed by a TBST wash. A working dye solution was applied and incubated in the dark at room temperature for 10 min, followed by another TBST wash. Subsequent antibody staining was performed by repeating antigen retrieval and subsequent steps, with primary antibodies for Tom1 (1:300, sc-514430, Santa Cruz Biotechnology, USA) and Tnnt2 (1:200, 15513-1-AP, Proteintech, Wuhan, China) applied sequentially. After completing the staining with all three primary antibodies, residual washing fluid was removed, DAPI working solution was used, and the sections were incubated at room temperature for 10 min, followed by a final TBST wash. Sections were then mounted with an anti-fade mounting medium and observed under a fluorescence microscope and photographed. The average fluorescence intensity of each group was analyzed using Image-J software.

### 2.6 Transmission electron microscopy

Under anesthesia, the rat hearts were rapidly taken, and the apex was cut and placed on a clean wax block in 4% paraformaldehyde, then immersed in 4% paraformaldehyde and fixed in a 4°C refrigerator for 48 h. After fixation, the tissues were washed with phosphate-buffered saline and dehydrated in a graded ethanol series. Pure acetone and embedding medium were prepared in a ratio of 1:1, and the myocardial tissue blocks were submerged for 90 min.

The infiltrated tissue blocks were then embedded in molds filled with embedding medium and heated in an oven at 60°C for 24 h to polymerize into blocks. Semi-ultrathin sections of 2 μm were cut, stained with methylene blue, and observed under a light microscope for positioning. Ultrathin sections of 50 nm were then cut and step-stained with saturated uranyl acetate and lead citrate for examination under a transmission electron microscope.

### 2.7 Real-time quantitative PCR (qRT-PCR)

Total RNA was extracted from myocardial tissues using the FstPure Cell/Tissue Total RNA Isolation Kit (RC101, Vazyme, Nanjing, China) according to the manufacturer's instructions, utilizing disposable, RNAse-free tips. Primer sequences were synthesized by AiKeRui Biological Engineering Co., Ltd., Hunan, China, as shown in Table 1. cDNA was synthesized using HiScript III RT SuperMix for qPCR (+gDNA wiper) (R323, Vazyme, Nangjing, China). The quantity of RNA in the cells was measured by adding Taq Pro Universal SYBR qPCR Master Mix (Q712, Vazyme, Nangjing, China), primers, and cDNA template. The qRT-PCR protocol included an initial denaturation at 95°C for 3 min, followed by amplification cycles of 95°C for 5 s and 60°C for 30 s, with a melting curve consisting of 95°C for 5 s, 60°C for 1 min, and a final extension at 60°C for 30 s. The relative expression levels of target genes were calculated using the 2^−*ΔΔ*Ct^ method (*Δ*Ct = target gene - GAPDH, *ΔΔ*Ct = *Δ*Ct experiment − *Δ*Ct control).

**Table 1:** Primeis sequences used for real-time PCR reactions.

Gene	Forward primer	Reverse primer
GAPDH	GGCACAGTCAAGGCTGAGAATG	ATGGTGGTGAAGACGCCAGTA
LC3-I	CATCGAGCGCTACAAGGGTGA	CGGATGATCTTGACCAACTCGC
LC3-Ⅱ	AGCTCTGAAGGCAACAGCAACA	GCTCCATGCAGGTAGCAGGAA
p62	GTTCCCAGAGGACGTGGTGTT	CACAGTGGCACCTCTGGTGAT
Beclin1	TTCCGTACAGGATGGACGTGG	CTTGAGCGCCTTTGTCCACTG
MYO6	AAAGCCGGAGGTGAACAGACA	TCTGCTCCCTCGTCATCATGG
Tom1	CTGCGCCATGAACGGTTTGAA	AAGGTCCACTCCTGCCAGTTG

### 2.8 Western Blotting (WB)

Approximately 50 mg of rat left ventricular tissue was weighed and used for protein extraction. Protein concentration was determined using the BCA method, and samples were denatured with loading buffer at 100°C for 10 min. Each lane was loaded with 40 μg of protein, separated on 10% and 12% gels by SDS-PAGE, and transferred to PVDF membranes. The membranes were blocked with 10% skim milk at room temperature for 1 h, then washed three times with TBST. Primary antibodies including GAPDH (1:10,000, AC001, ABclonal Technology, Hubei, China)), LC3 (1:1,000, ab192890, Abcam, UK), p62 (1:1,000, ab109012, Abcam, UK), Beclin1 (1:1,000, 11306-1-AP, Proteintech, Wuhan, China), MYO6 (1:500, 26778-1-AP, Proteintech, Wuhan, China), and Tom1 (1:500, sc-514430, Santa Cruz Biotechnology, USA) were added and incubated overnight at 4°C. After incubation, corresponding secondary antibodies were added and incubated at room temperature for 1 h. The membranes were washed three times with TBST and developed using the ECL method. GAPDH was used as an internal control. The gray value of each band was measured using Image-J software.

### 2.9 Docking experiment

Protein Docking of MYO6 and Tom1: The protein structure of MYO6 and Tom1 was downloaded from the UniProt database, and the interaction mode of MYO6 and Tom1 was studied by Hdock. PyMOL 2.3.0 is used to analyze the interaction mode of the docking result.

Molecular docking experiments of MYO6-Tom1 protein complex with AR-DS major components: Small molecule design and processing: Download the 3D structure in SDF format from PubChem data according to the CAS number of the small molecule, import the structure into ChemBio3D Ultra 14.0 for energy minimization, set the Minimum RMS Gradient to 0.001, and save the small molecule in mol2 format. The optimized small molecules were imported into AutodockTools-1.5.6 for hydrogenation, charge calculation, charge assignment, and setting of rotatable keys, and then saved as “pdbqt” format. Preparation and processing of protein: Use PyMOL 2.3.0 to remove protein water of crystallization, original ligand, etc. Import protein structure into AutoDocktools (v1.5.6) for hydrogenation, charge calculation, charge assignment, atom type assignment, and save it in “pdbqt” format. Preparation of parameter files: protein binding sites were predicted using POCASA 1.1, docking was performed using AutoDock Vina 1.1.2, and protein-related parameters were set to: center_*x* = 20.7, center_*y* = 13.9, center_*z* = −33.1; search space: size_*x*:60, size_*y*: 60. size_*z*:60 (each grid point is spaced at 0.375 Å), exhaustiveness: 10, and the rest of the parameters are set by default. The results were analyzed for interaction patterns using PyMOL 2.3.0 for the docking results.

### 2.10 Statistical analysis

The data were presented as mean ± standard error of the mean (SEM). A statistically significant difference was determined by *p* < 0.05. The means between the two groups were analyzed using a two-tailed Student's t-test. One-way analysis of variance (One-way ANOVA) was utilized for multiple comparisons involving more than two groups.

## 3 Results

### 3.1 Analysis of the active components of AR-DS by UPLC-Q-Exactive-MS

**Figure 1:**
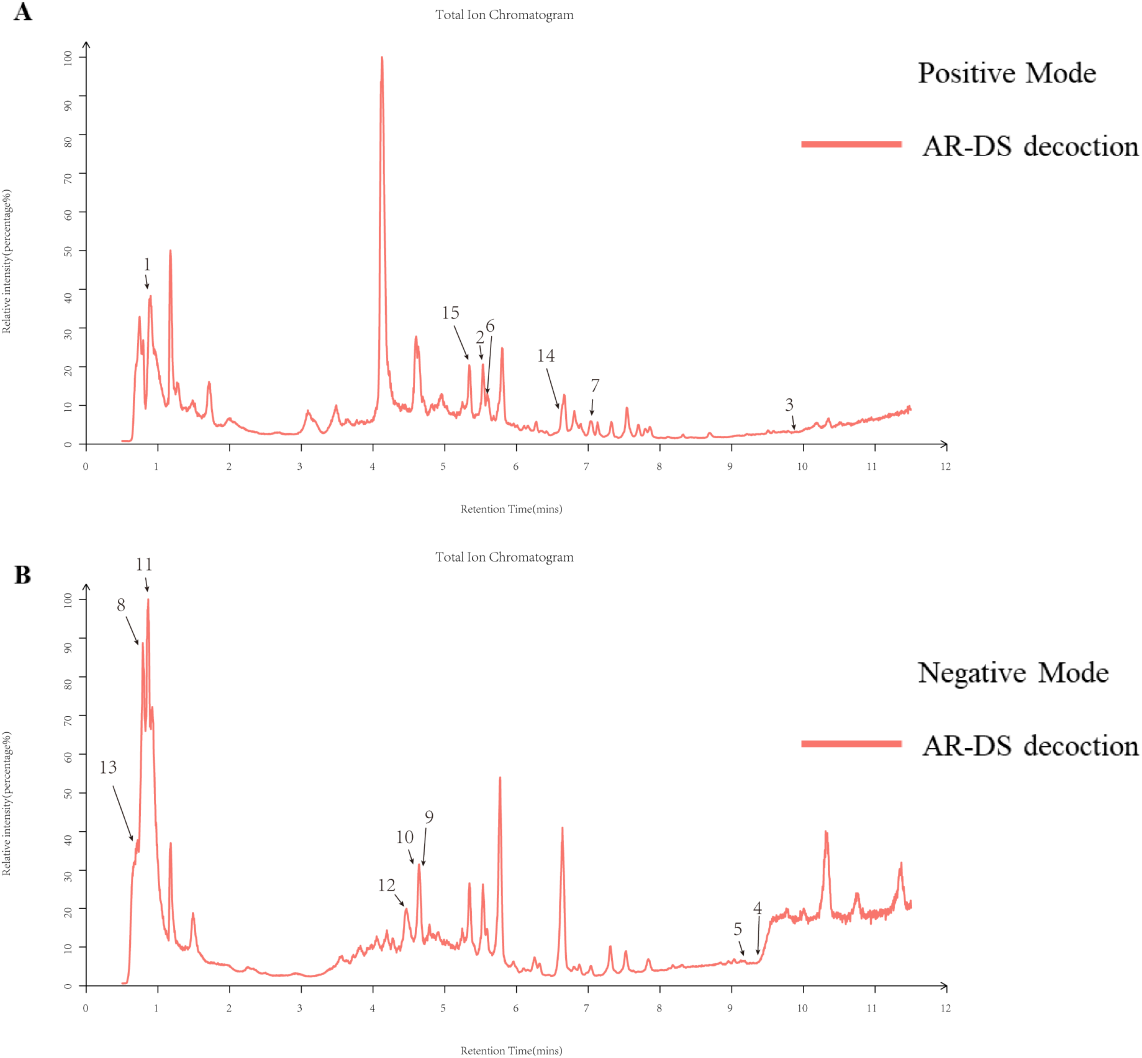
Total ion flow diagrams for natural product identification in AR-DS aqueous decoction. **(A)** Total ion flow diagram for positive ion mode. **(B)** Total ion flow diagram for negative ion mode.

The chemical composition of AR-DS was analyzed by UPLC-Q-Exactive-MS/mass spectrometry. Human Metabolome Database (HMDB) (http://www.hmdb.ca) and massbank (http://www.massbank.jp/) l,LipidMaps) are used for substance identification (http://www.lipidmaps.org1, mzclound (https://www.mzcloud.org) spectra database retrieval comparison, and contrast (42–45) with the existing literature. Figures 1A, B are the UPLC positive ion peak and negative ion peak of AR-DS aqueous solution, respectively. We labeled the main chemical components contained in AR-DS. As is shown in Table 2, fifteen components were identified as L-Arginine, Daidzein, Beta-Sitosterol, Thymidine, Quercetin, Kaempferol, Ononin, Mannitol, Folic acid, Astragaloside IV, L-Threonine, Rutin, Formononetin, S-Adenosylmethionine, and Isorhamnetin.

**Table 2:** LC-MS analysis of AR-DS aqueous decoctions.

No.	Name	RT (sec)	Formula	CAS	MW	Measured value	PPM	Fragment ions	Remark	From
1	L-Arginine	54.6	C_6_H_14_N_4_O_2_	74-79-3	174.2	175.0717	6.530806378	60.06, 70.07, 116.07, 175.12	[M + H]^+^	AR
2	Daidzein	336.8	C_15_H_10_O_4_	486-66-8	254.24	255.0651	0.297963147	255.07	[M + H]^+^	AR
3	Beta-Sitosterol	594	C_29_H_50_O	83-46-5	414.7	397.3807	4.866869478	81.07, 95.09, 147.12	[M + H-H2O]^+^	DS
4	Thymidine	560.5	C_10_H_14_N_2_O_5_	50-89-5	242.23	223.0293	6.311355275	223.03	[M-H2O-H]^−^	AR
5	Quercetin	549.7	C_15_H_10_O_7_	117-39-5	302.23	301.1675	14.12147622	301.1	[M-H]^−^	AR, DS
6	Kaempferol	340.8	C_15_H_10_O_6_	520-18-3	286.24	287.0552	0.780337719	56.05, 72.04, 99.06, 114.07, 127.11, 147.04, 153.06, 160.07, 177.06, 255.07, 268.17, 286.18, 288.19	[M + H]^+^	DS
7	Ononin	429.6	C_22_H_22_O_9_	486-62-4	430.4	430.1313	11.39187034	671.42, 689.43, 691.43, 707.44, 731.61, 758.57, 798.55, 812.57, 833.47, 851.48, 869.49, 871.50, 891.47, 914.55	[M]^+^	AR
8	Mannitol	49.9	C_6_H_14_O_6_	69-65-8	182.17	181.0723	0.105572255	111.0094, 129.0201, 141.0202, 159.0307, 177.0414, 195.0520	[M-H]^−^	AR
9	Folic acid	276.9	C_19_H_19_N_7_O_6_	59-30-3	441.4	440.1311	2.719510932	111.2834, 127.1135, 153.0928, 171.1035	[M-H]^−^	AR
10	Astragaloside IV	273.1	C_41_H_68_O_14_	84687-43-4	785	783.4539	0.343499471	134.0385, 150.0340, 175.0410, 193.0517	[M + CH3COO]^−^	AR
11	L-Threonine	56.8	C_4_H_9_NO_3_	72-19-5	119.12	118.0511	1.490879797	118.0517, 140.3700	[M-H]^−^	AR
12	Rutin	265.5	C_27_H_30_O_16_	153-18-4	610.5	609.146	0.203563678	113.0246, 134.0381, 149.0618, 161.0459, 175.0409, 193.0516, 205.0505, 223.0618	[M-H]^−^	AR
13	Formononetin	45.7	C_16_H_12_O_4_	485-72-3	268.264	268.0766	11.19083128	57.5628, 100.0411, 118.0515, 133.0175, 151.0285, 166.0518, 176.3635, 208.0991, 226.1565, 269.2131	[M]^−^	AR
14	S-Adenosylmethionine	398.2405	C_15_H_22_N_6_O_5_S	17176-17-9	453.6	398.137	0.806396	398.24	[M]^+^	DS
15	Isorhamnetin	317.0658	C_16_H_12_O_7_	480-19-3	229.2	316.0583	0.706478	317.07	[M + H]^+^	DS

### 3.2 The effect of AR-DS on echocardiographic parameters in heart failure rats

**Figure 2:**
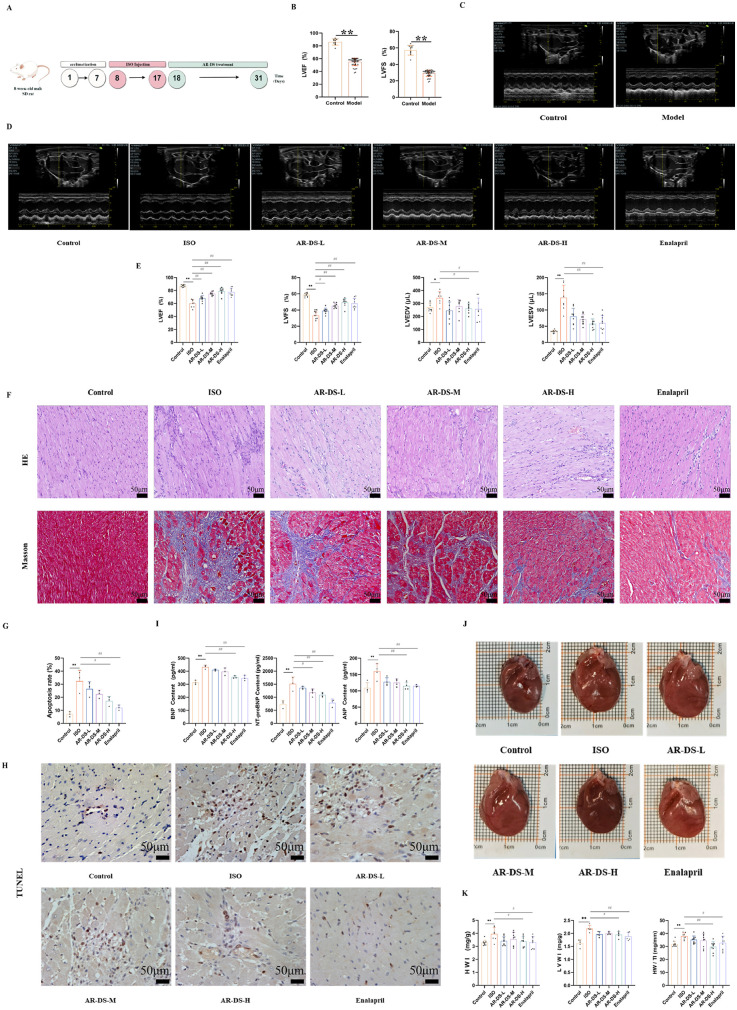
Specific experimental design and implementation **(A)** ultrasound detection results of rats after 10 days of intervention by ISO, 10 rats in the control group, and *n* = 44 rats in the model group **(B,C)**. Ultrasonography results after intervention with drugs in each group of rats **(D,E)**. Comparison with Control group: **P < 0.01, *P < 0.05; Comparison with ISO group: ^##^*P* < 0.01, ^#^*P* < 0.05, 8–10 rats in each group. HE and Masson staining of rat myocardium in each group (40×, scale bar = 50 μm). **(F)** Myocardial apoptosis rate statistics **(G)** and TUNEL staining (40×, scale bar = 50 μm) of rats in each group **(H)** Serum BNP, NT-pro BNP, and ANP levels in rats in each group **(I)**. Effects of rat heart and heart mass-related indices in each group of rats **(J,K)**. Comparison with Control group: **P < 0.01, Comparison with ISO group: ^##^*P* < 0.01, ^#^*P* < 0.05, *n* = 3.

Male SD rats were acclimatized and fed for 7 days, and after 10 days of isoprenaline hydrochloride solution injection, LVEF and LVFS were evaluated by M-mode echocardiography, and those with LVEF less than 60% and LVFS less than 35% (38–41) were selected as rats with successful model replication of the heart failure model as shown in Figures 2B,C.

The rats in the treatment group were given the drug by gavage for 14 days, and equal amounts of saline were given to the control and ISO groups. M-mode echocardiography was used to evaluate LVEF, LVFS, left ventricular end-diastolic volume (LVEDV), and left ventricular end-systolic volume (LVESV) to observe changes in cardiac function. Compared to the Control group, rats in the isoprenaline hydrochloride solution-induced heart failure model showed significantly reduced cardiac contractile function, as indicated by decreased LVEF (*P* < 0.01) and LVFS (*P* < 0.01), and increased LVEDV (*P* < 0.05) and LVESV (*P* < 0.01). After 14 days of treatment, compared to the ISO group, rats in the AR-DS-H group showed a significant decrease in LVEDV (*P* < 0.05) and LVESV values (*P* < 0.01) and significant increases in LVEF (*P* < 0.01) and LVFS (*P* < 0.01), as shown in Figures 2D,E.

### 3.3 The effect of AR-DS on cardiac hypertrophy and levels of BNP, NT-pro BNP, and ANP in heart failure rats

Compared to the Control group, rats in the ISO group exhibited significant cardiac hypertrophy, which was ameliorated following treatment with AR-DS. As shown in Figure 2J, compared to the Control group, the heart weight index (HWI), left ventricular weight index (LVWI), and heart-to-tibia length ratio (HW/TI) in the ISO group were significantly elevated (*P* < 0.01). Compared to the ISO group, the AR-DS-H group showed a significant reduction in HWI (*P* < 0.05), LVWI (*P* < 0.05), and HW/TI (*P* < 0.01), as shown in Figure 2K. Compared to the Control group, the heart function-related biomarkers BNP, NT-pro BNP, and ANP were significantly elevated in the serum of rats in the ISO group (*P* < 0.01). Treatment with AR-DS-H significantly improved the cardiac status of heart failure rats (*P* < 0.01), as shown in Figure 2I.

### 3.4 AR-DS significantly improves cardiac tissue pathology and apoptosis levels in heart failure rats

HE staining results showed that compared to the Control group, myocardial cells in the ISO group were disorganized, with increased interstitial cells and inflammatory cell infiltration. In comparison to the ISO group, the treatment groups showed a marked reduction in myocardial hypertrophy, more organized cell alignment, and less noticeable increases in interstitial cells. Masson staining revealed that compared to the Control group, rats in the ISO group exhibited coarser collagen fibers, disordered cell alignment, and increased deposition of myocardial tissue collagen. After drug intervention, compared to the ISO group, the collagen fibers in rats became finer, and cell alignment became more orderly. As shown in Figure 2F, TUNEL staining indicated that myocardial cell apoptosis was exacerbated in the ISO group compared to the Control group (*P* < 0.01), and AR-DS treatment significantly improved the apoptosis (*P* < 0.05), as shown in Figures 2G,H.

### 3.5 Effect of AR-DS on myocardial autophagy in rats with heart failure

**Figure 3:**
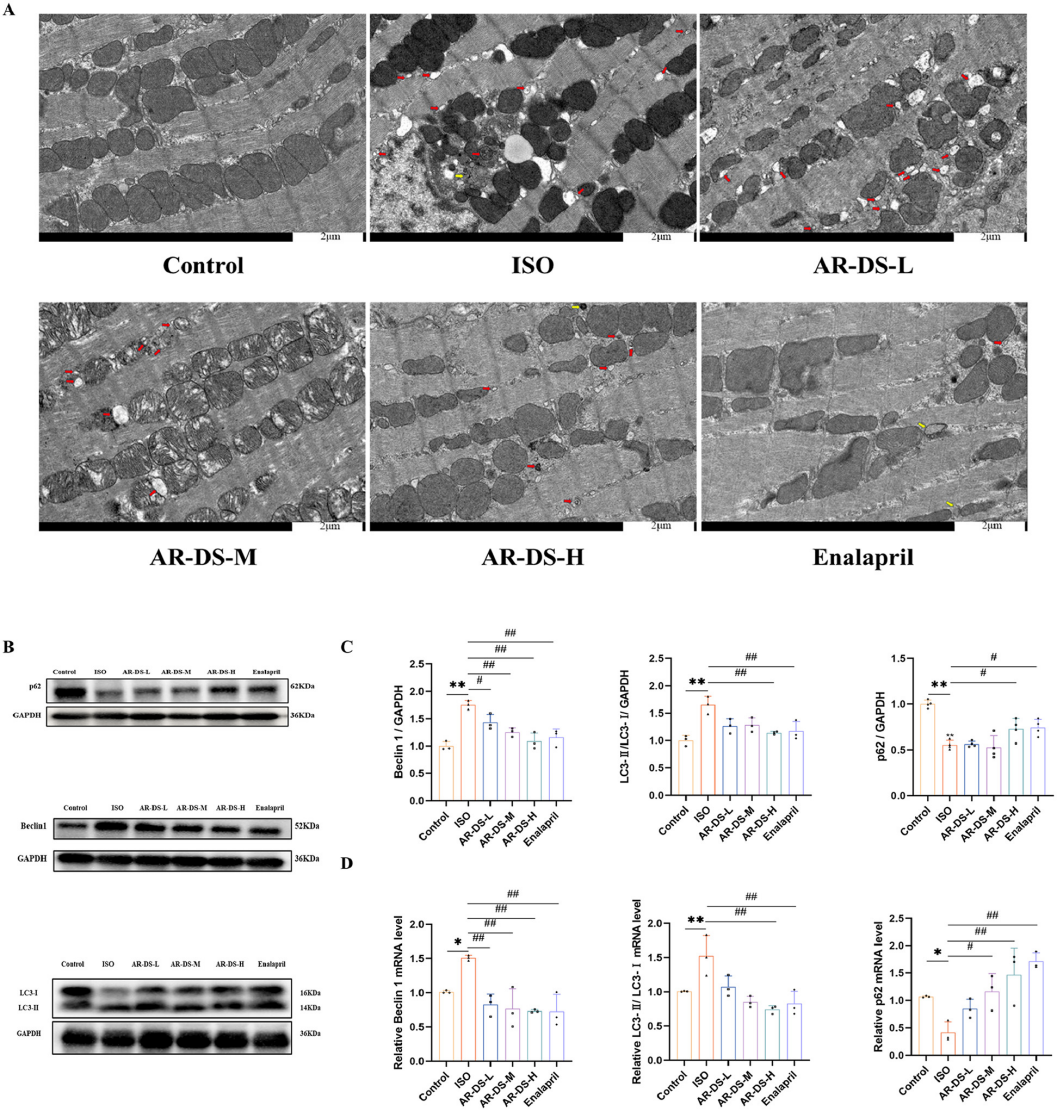
Comparison of myocardial ultrastructure across groups. Red arrows indicate autophagosomes, and yellow arrows point to autolysosomes. **(A)** Relative protein expression and statistics of p62, Beclin1, and LC3 in the myocardium of rats in each group **(B,C)**. Relative gene expression and statistics of p62, Beclin1, and LC3 in the myocardium of rats in each group **(D)** Compared to the Control group: **P < 0.01, *P < 0.05; compared to the ISO group: ^##^*P* < 0.01, ^#^*P* < 0.05, *n* = 3.

Transmission electron microscopy results showed that compared to the Control group, rats in the ISO group had mitochondria that were swollen, dissolved, and increased in number and aggregation, with numerous autophagosomes and a few autolysosomes forming. Compared to the ISO group, both the AR-DS-H and Enalapril groups showed a significant reduction in the number of autophagosomes, a noticeable decrease in mitochondrial numbers, and an alleviation of mitochondrial aggregation, as illustrated in Figure 3A.

To explore the specific mechanism through which AR-DS inhibits heart failure, we first examined the protein expression levels of LC3, p62, and Beclin1. Figures 3B,C show the expression levels of LC3, p62, and Beclin1 proteins. Compared to the Control group, expression levels of LC3 and Beclin1 proteins were increased in the ISO group (*P* < 0.01), while the expression level of the p62 protein was decreased (*P* < 0.01). After treatment with AR-DS-H, there was a significant downregulation of LC3 and Beclin1 protein expression (*P* < 0.01) and an upregulation of p62 protein expression (*P* < 0.05). Further validation of the expression levels of LC3, p62, and Beclin1 genes in the myocardium yielded results consistent with the protein levels. Compared to the Control group, expression levels of LC3 (*P* < 0.01) and Beclin1 (*P* < 0.05) genes were increased in the ISO group, while the expression level of the p62 gene was decreased (*P* < 0.05). After treatment with AR-DS-H, there was a significant downregulation of LC3 and Beclin1 protein expression (*P* < 0.01) and an upregulation of p62 protein expression (*P* < 0.01). As shown in Figure 3D.

### 3.6 AR-DS modulates the expression of MYO6 and Tom1 in Rat myocardium

**Figure 4:**
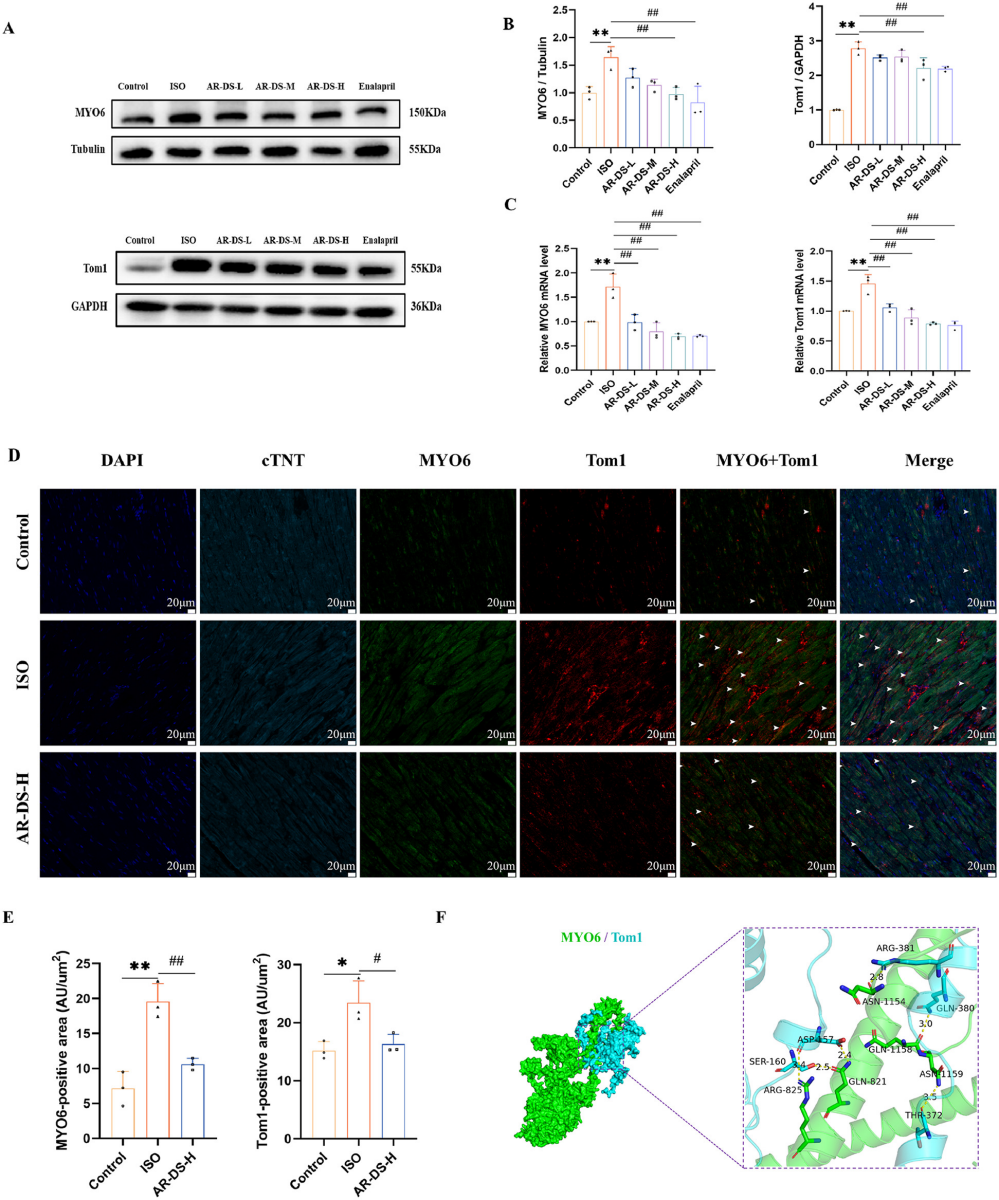
Relative protein expression and statistics of MYO6 and Tom1 in rat myocardium in each group **(A,B)**. Relative gene expression and statistics of MYO6 and Tom1 in rat myocardium in each group **(C)** Multiple immunofluorescence staining images of rat myocardium in the Control, ISO, and AR-DS-H groups. MYO6 and Tom1 co-expressing cells were labeled using white arrows. (60×, scale bar = 20 μm) **(D)** Analysis of the average fluorescence intensity of MYO6 and Tom1 in the myocardium of rats from the Control, ISO, and AR-DS-H groups **(E)** Compared to the Control group: **P < 0.01, *P < 0.05; compared to the ISO group: ^##^*P* < 0.01, ^#^*P* < 0.05, *n* = 3. Protein docking image of MYO6 and Tom1, where green represents the MYO6 protein and sky blue represents the Tom1 protein **(F)**

Research indicates that during autophagy, impairment in the binding of MYO6 to Tom1 can block the fusion of autophagosomes with lysosomes (27, 29), so the MYO6-Tom1 complex may affect the process of autophagy. As shown in Figures 4A,B, western blot results revealed that compared to the Control group, the protein expressions of MYO6 and Tom1 were significantly increased (*P* < 0.01). After treatment with AR-DS, the AR-DS-H group showed a significant decrease in the protein expression of MYO6 and Tom1 (*P* < 0.01). As depicted in Figure 4C, qRT-PCR results indicated a significant increase in the gene expressions of MYO6 and Tom1 (*P* < 0.01). After treatment with AR-DS, the AR-DS-H group exhibited a significant decrease in the gene expression of MYO6 and Tom1 (*P* < 0.01).

We selected the Control group, the ISO group, and the AR-DS-H group for analysis through multiple immunofluorescence staining to observe the protein expression of MYO6 and Tom1. As shown in Figure 4E, MYO6 (*P* < 0.01) and Tom1 (*P* < 0.05) were highly expressed in the ISO group, while AR-DS-H significantly downregulated the protein expression of both MYO6 (*P* < 0.01) and Tom1 (*P* < 0.05).

To further explore the binding capacity of MYO6 and Tom1, protein docking was performed. As illustrated in Figure 4F, the docking results indicated that the binding energy between MYO6 and Tom1 was −256.89 kcal/mol. The residues surrounding the protein-protein interaction interface could form hydrogen bonds, potentially playing an active role. Specifically, residues ARG-825, GLN-821, ASN-1159, GLN-1158, and ASN-1154 of MYO6 formed hydrogen bonds with ASP-457, SER-160, THR-372, GLN-380, and ARG-381 of Tom1, respectively. The lengths of these hydrogen bonds were 3.4 Å, 2.5 Å, 2.4 Å, 3.5 Å, 3.0 Å, and 2.8 Å, which help stabilize the protein-protein complex.

**Figure 5:**
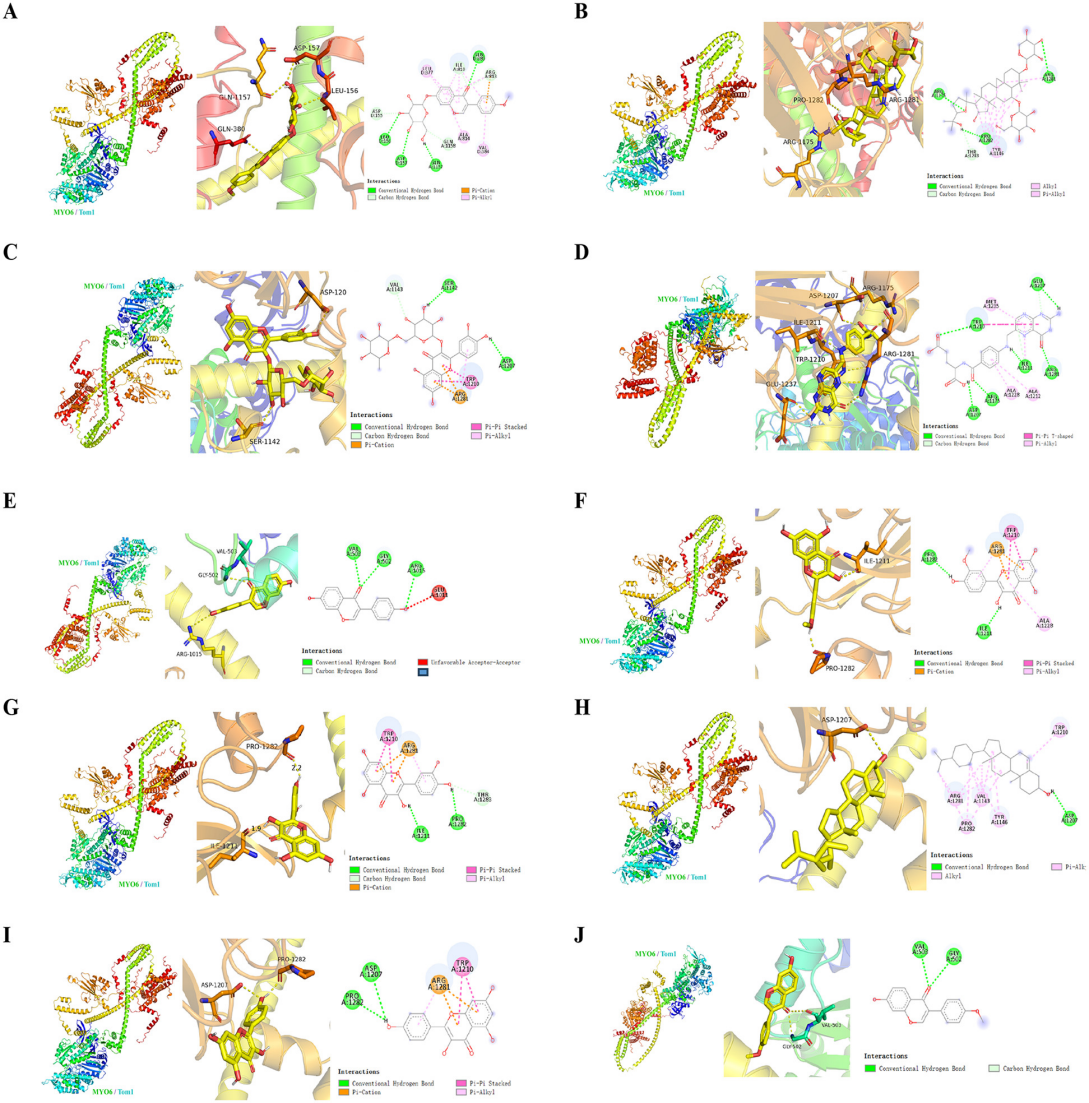
Mode of action, 3D, active pocket, and 2D maps of 10 compounds in MYO6-Tom1 complex and AR-DS decoction. The compounds were Ononin **(A)**, Astragaloside-IV **(B)**, Rutin **(C)**, Folic-acid **(D)**, Daidzein **(E)**, Isorhamnetin **(F)**, Quercetin **(G)**, Beta-Sitosterol **(H)**, Kaempferol **(I)**, Formononetin **(J)**.

Finally, to predict the possible sites of AR-DS action on MYO6-Tom1, we molecularly docked the MYO6-Tom1 complex with AR-DS. As shown in Figure 5. It was found that Ononin, Astragaloside-IV, Rutin, Folic-acid, Daidzein, and Formononetin derived from AR, and Isorhamnetin, Quercetin, Beta-Sitosterol, and Kaempferol derived from DS exhibited strong binding ability, as shown in Table 3. It further suggests that AR-DS may exert its interfering effects through these sites.

**Table 3:** Binding affinity and binding site between the MYO6-Tom1 protein complex and the AR-DS components.

No.	Component	Binding affinity (kcal/mol)	Binding site	From
1	Ononin	−8.8	GLN1157, ASP157, LEU156, GLN580, VAL384, ALA814, LEU377, ILE810, ASP155, GLN1158, ARG813	AR
2	Astragaloside-IV	−8.6	ARG11175, PRO1282, ARG1281, TYR1146, THR1283	AR
3	Rutin	−8.6	ARG11175, PRO1282, ARG1281, TYR1146, THR1283	AR
4	Folic-acid	−7.8	ASP1207, ARG1175, ILE1211, ARG1281, GLU1237, TRP1210, ALA1228, ALA1212, ILE1211, TRP1210	AR
5	Daidzein	−7.6	ASP1207, ARG1175, ILE1211, ARG1281, GLU1237, TRP1210, ALA1228, ALA1212, ILE1211, TRP1210	AR
6	Isorhamnetin	−7.6	ILE1211, PRO1282, ALA1228, ARG1281, TRP1210, ARG1281	DS
7	Quercetin	−7.6	ILE1211, PRO1281, ARG1281, TRP1210, ARG1281	AR, DS
8	Beta-Sitosterol	−7.5	ASP1207, TRP1210, TYR1146, VAL1143, PRO1282, ARG1281	DS
9	Kaempferol	−7.5	PRO1281, ASP1207, TRP1210, ARG1281	DS
10	Formononetin	−7.3	VAL503, GLY502, GLY502	AR

## 4 Discussion

Heart failure is a syndrome caused by various heart diseases such as coronary artery disease, myocardial ischemia, and hypertensive heart disease. It is characterized by structural and functional abnormalities of the heart, leading to chamber enlargement and contractile and/or diastolic dysfunction. Currently, over 64 million (46) people worldwide suffer from heart failure, posing a serious threat to human health. We constructed a rat model of heart failure by subcutaneously injecting isoprenaline hydrochloride solution to explore new therapies for heart failure (35–37).

To analyze the anti-heart failure components contained in AR-DS, we employed UPLC-Q-Exactive-MS/ mass spectrometry to analyze the components of the AR-DS decoction. AR is commonly used in TCM for treating heart failure. Modern pharmacological studies indicate that AR has effects such as improving heart function, regulating blood pressure, and reducing inflammatory responses (47). Studies by Wang et al. (48) found that Astragaloside IV can increase the survival rate of doxorubicin-induced heart failure rats and enhance myocardial contractility. Research by Li et al. (49) discovered that Daidzein can increase the expression of SERCA mRNA, decrease the expression of PLB mRNA, and improve the PLB/SERCA ratio, effectively ameliorating heart failure. Wang et al. (50) found that L-Arginine significantly improves vascular endothelial function in patients with heart failure. DS has been shown to effectively improve heart function (51), with components like Quercetin, Kaempferol, and Isorhamnetin—rare in nature but abundant in DS, acting as characteristic components (52). Studies by Yu et al. (42, 43) identified the main effective components against heart failure in AR-DS as Quercetin, Kaempferol, Isorhamnetin, Beta-Sitosterol, Formononetin, and Folic acid. Our findings confirm the presence of these components in the AR-DS decoction.

TCM's earliest references to “heart failure” can be found in the *Mai Jing* from the Western Jin dynasty. Clinically, heart failure was categorized under symptoms such as palpitations, edema, asthma, and “heart water.” The fundamental pathology of this disease is rooted in Qi deficiency, with the symptom characterized by the accumulation of retained fluid. Often a result of chronic heart disease, it leads to insufficient heart Qi. Firstly, this insufficiency causes the heart to lack the necessary support, leading to a sense of uneasiness, which may manifest as palpitations. Furthermore, insufficient heart Qi disrupts the transformation and transportation of Qi, leading to the retention of fluid internally. Taking advantage of the vulnerability of heart Qi, the retained fluid overwhelms and ascends to the heart and lungs, thus triggering heart failure. The appropriate treatment should focus on enhancing Qi and facilitating diuresis. A combination of Qi-boosting and diuresis-facilitating drugs is a common treatment in clinical prescriptions for heart failure. Clinical studies have shown that AR-DS is the most frequently used herbal pair for boosting Qi and promoting diuresis (14, 15). Meanwhile, clinical studies have found that patients with heart failure with diuretic resistance have a significantly higher readmission rate within 60 days after treatment with Western drugs alone (53). Diuretic resistance becomes a challenge in the management of heart failure (54, 55). Studies have found that TCM therapies show advantages in improving diuretic resistance (56). AR exerts a beneficial effect on qi and at the same time improves blood circulation to the kidneys, increasing renal blood flow and providing more raw materials for urine production (57). DS has the effect of enhancing cardiac function. It also inhibits cardiac secretion of BNP and exerts positive inotropic effects, while promoting diuresis by lowering the concentration of sodium ions in the serum (13). AR and DS work synergistically to promote the elimination of excess water from the body, reduce water and sodium retention, and relieve symptoms of diuretic resistance (56). Therefore, AR-DS plays a unique role in the treatment of diuresis and enhancement of cardiac function in heart failure.

Echocardiography is a widely used method for assessing cardiac function. LVEF measures the ejection capacity of the left ventricle, while LVFS reflects the degree of left ventricular contraction; both indices evaluate the systolic and diastolic functions of the left ventricle (58). This study found significant improvements in cardiac function in the AR-DS-treated group compared to the ISO group, as evidenced by increased LVEF and LVFS, and reduced LVEDV and LVESV, particularly in the high-dose group. Echocardiographic results confirmed that AR-DS-H effectively protects myocardial function in heart failure. The physical changes in organs directly reflect the pathological alterations in heart failure rats. Following the induction of the heart failure model, there was a noticeable enlargement and mass increase of the heart, with thickening most apparent in the left ventricle. The rise in the HW/TI also indicated myocardial hypertrophy. Our analysis of HWI, LVWI, and HW/TI showed that the AR-DS-H was effective in reducing all three indices. The severity of cardiac tissue damage is most directly reflected through histomorphology; thus, we conducted further observations on the cardiac tissue pathology. HE and Masson's staining results indicated that AR-DS treatment effectively ameliorated issues of myocardial cell hypertrophy, inflammatory infiltration, and myocardial fiber deposition. Heart failure is also accompanied by changes in related hormones. Our analysis of serum levels of BNP, NT-pro BNP, and ANP in rats revealed significant increases in the ISO group compared to the Control group, which were notably reduced after treatment with AR-DS-H. These results all demonstrate the reliable efficacy of AR-DS in treating heart failure in rats.

Research indicates that excessive activation of autophagy is a significant factor leading to heart failure (59). Autophagy is a highly conserved intracellular lysosome-mediated mechanism involved in the degradation of proteins and organelles, and a certain degree of autophagy plays an important role in maintaining homeostasis in the cardiovascular system (19). In the basal state, cellular autophagy plays an anti-apoptotic and cytoprotective role to maintain cardiomyocyte homeostasis. Over-activated autophagy results in the accumulation of numerous autophagosomes, autolysosomes, and vacuoles (60), and can exacerbate cardiomyocyte apoptosis (20) along with a series of myocardial pathological changes (21). LC3 is a classical marker of autophagosomes during autophagy, where the conversion of LC3-I to LC3-II and its recruitment to autophagosomes are critical steps in autophagosome formation. Thus, the level of LC3-II reflects the number of autophagosomes and autophagy-related structures (61); p62, a substrate of LC3-II, increases in expression when autophagy levels decrease (62). Beclin1 is an important regulatory gene involved in autophagy, and its expression level is highly correlated with autophagic activity (63). Transmission electron microscopy results showed that compared to the Control group, the ISO group exhibited a significant increase in autophagosomes and autolysosomes in myocardial cells. There was an upregulation of protein and gene levels of LC3 II/I and Beclin1, and a downregulation of p62, indicating significant activation of autophagy. After AR-DS treatment, there was a noticeable reduction in autophagosomes and autolysosomes, downregulation of LC3 II/I and Beclin1, upregulation of p62, and a reduction in the rate of cardiomyocyte apoptosis, significantly decreasing the level of myocardial autophagy.

The transport process of autophagy relies on the cytoskeleton (22), which is a spatial network within cells composed of fibrous proteins, including microtubules, microfilaments, and intermediate filaments. Microfilaments serve as the dynamic foundation for the occurrence and development of autophagy, providing a fluid fibrous network that facilitates the transport of membrane structures to autophagosomes across different cellular regions (64). This fibrous network structurally supports the expansion of autophagic vacuoles (65), the movement of autophagosomes, and their effective fusion with lysosomes (66). Myosin, bound to microfilaments, mediates cellular movement or material transport through its functional domains, which include a motor domain, a regulatory domain, and a tail domain. The motor domain, located at the head of myosin, contains an actin-binding site and an ATP-binding site with ATPase activity. Traditionally, when the motor domains of myosin heads bind to ATP, their affinity for microfilaments decreases, enabling myosin to bind to the surface of microfilaments and move towards the positive end of the microfilament (67–70). MYO6 is the only unconventional myosin motor within eukaryotic cells that moves towards the negative end of the microfilament on its surface. Due to its unique reverse gear motion, MYO6 plays various unique cellular functions, ranging from vesicle transport and clathrin-mediated endocytosis to roles in autophagy and cell migration (25–28). Studies have suggested that mutations in the MYO6 gene might be associated with arrhythmias and episodes of sudden cardiac death (71), indicating its involvement in the regulation of cardiac growth and function. The absence of MYO6 may lead to cardiac dysfunction (72). Research has found (73) that Myosin Heavy Chain 6 expression levels are elevated in the hearts of rats with an isoproterenol-induced cardiomyopathy model, and that Astragaloside IV combined with DS downregulates Myosin Heavy Chain 6 expression in the myocardium. Through WB and qRT-PCR, we detected MYO6 protein and gene expression in the myocardium, finding significant increases in the ISO group and notable downregulation following treatment with AR-DS.

Research has discovered (24) that the functional diversity associated with MYO6 stems from its interactions with multiple transport adaptors. The C-terminal of MYO6 contains a cargo-binding domain with a WWY motif. During cellular autophagy, MYO6 specifically binds to the adaptor protein Tom1 via the WWY sequence in the cargo-binding domain, mediating its connection to endosomes and targeting lysosomes (74). This interaction forms the MYO6-Tom1 complex, allowing endosomes connected to Tom1 to come into close contact with autophagosomes via MYO6, forming intermediary vesicles. This promotes the maturation of autophagosomes, drives fusion with lysosomes, and triggers autophagic flux (75). We utilized WB to detect the expression of Tom1 in myocardial tissue, finding that Tom1 expression was elevated in the ISO group. Following treatment with AR-DS, Tom1 expression was effectively downregulated. Subsequent qRT-PCR analysis of Tom1 gene expression confirmed that changes at the genetic level were consistent with those observed at the protein level.

Research has confirmed that the interaction between MYO6 and its adaptor protein Tom1 facilitates the transport of Tom1-positive nucleosomes across the endosomal membrane to autophagosomes, thereby promoting autophagosome maturation and fusion with lysosomes. Therefore, blocking the MYO6-Tom1 interaction can impede the fusion of autophagosomes with lysosomes (24, 29, 75). We first observed the expression of MYO6 and Tom1 in myocardial tissue using multiple immunofluorescent stainings. By measuring the average fluorescence intensity of MYO6 and Tom1, we observed that AR-DS treatment effectively reduced their expression. Using cardiac troponin T to mark the cardiomyocytes, we noted higher expression of MYO6 in the myocardial interstitium. Studies have shown that MYO6 is primarily expressed in vascular endothelial cells within mouse hearts (76), which provides clues for further exploration of the role of MYO6 in different cardiac cell types. The binding efficiency of MYO6 with Tom1 affects the fusion of autophagosomes with lysosomes, which is crucial for the MYO6-Tom1 complex's impact on autophagy (29, 75). Therefore, we performed protein docking between MYO6 and Tom1 and found a binding energy of −256.89 kcal/mol. The residues surrounding the protein-protein interaction interface could form hydrogen bonds. These non-covalent bonds help stabilize the MYO6-Tom1 complex, indicating that MYO6 and Tom1 can form a stable complex.

To further explore the possible sites of AR-DS regulation of the MYO6-Tom1 complex, we molecularly docked the MYO6-Tom1 complex with AR-DS. It was found that Ononin, Astragaloside-IV, Rutin, Folic-acid, Daidzein, Formononetin, Isorhamnetin, Quercetin, Beta-Sitosterol, and Kaempferol all exhibited strong binding. This suggests that AR-DS is likely to regulate the MYO6-Tom1 complex through these components. This also points out the direction for our next research. Therefore, AR-DS may inhibit heart failure in rats by blocking the formation of the MYO6-Tom1 complex in the myocardium, thereby impeding the fusion of autophagosomes with lysosomes and reducing myocardial autophagy, ultimately improving cardiac function in heart failure rats.

**Figure 6:**
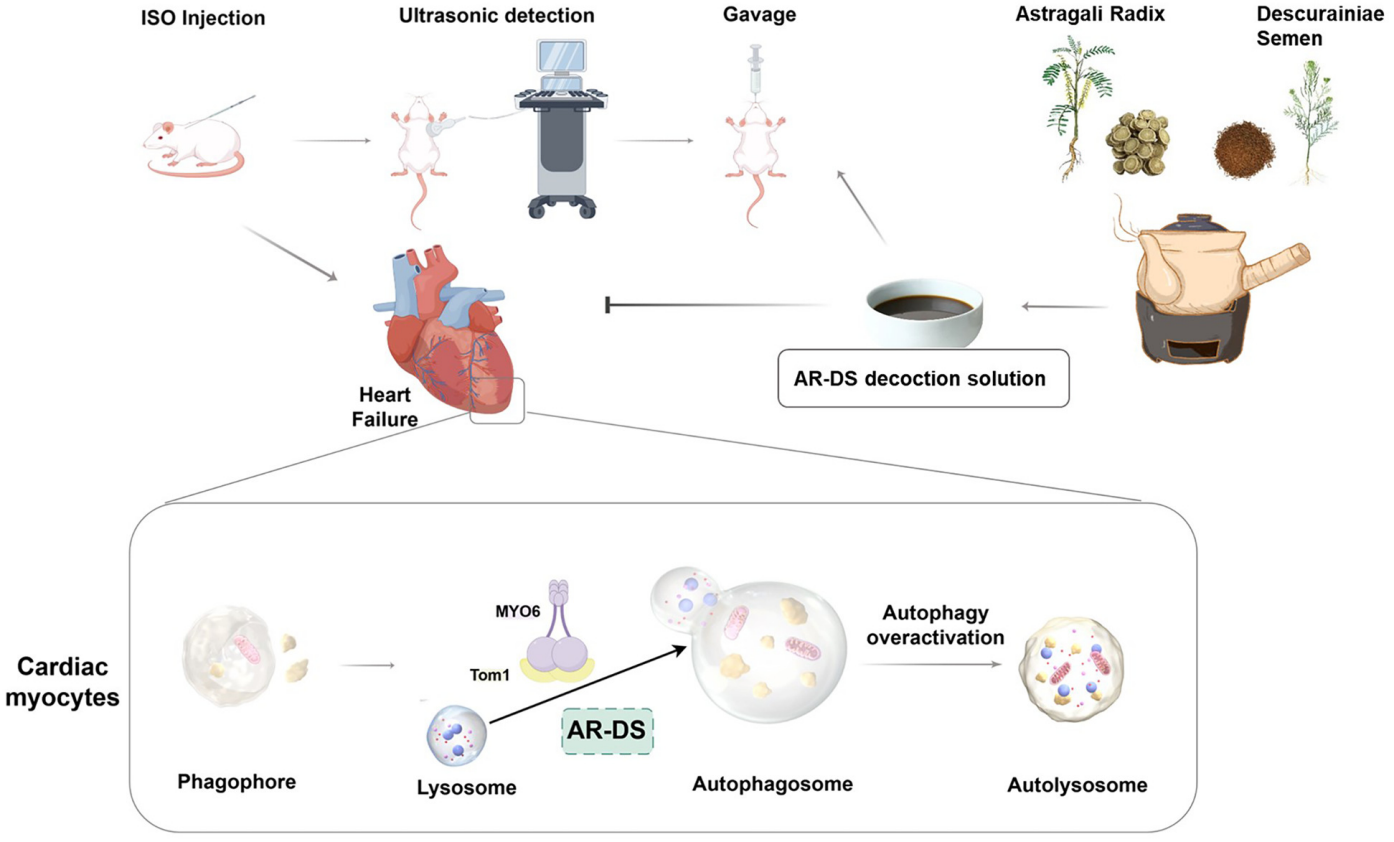
AR-DS may regulate myocardial autophagy in HF rats through the MYO6-Tom1 complex. (Created using Figdraw).

In summary, our study has for the first time demonstrated that the potential mechanism by which AR-DS treats heart failure may involve the inhibition of myocardial autophagy through the MYO6-Tom1 complex. Figure 6 illustrates the overall process following administration. Additionally, there are many limitations of this experimental study. We only elucidated the mechanism of action of AR-DS from an *in vivo* perspective, so in the future, we will work on clarifying the types of cardiomyocytes in which AR-DS acts and elucidating the mechanism of action of AR-DS on MYO6 from an *in vitro* perspective, to enrich the argumentation for AR-DS. Meanwhile, although we predicted the site of action of the main component of AR-DS on MYO6-Tom1, we did not construct a corresponding model to verify it, which is also the direction of our next efforts.

## 5 Conclusions

This study found that AR-DS inhibits overly activated autophagy by regulating the MYO6-Tom1 complex. It, thereby, halts the progression of heart function deterioration, partially elucidating its pharmacological mechanism against heart failure. This provides a more solid theoretical basis for its application in the treatment of heart failure.

## Data Availability

The original contributions presented in the study are included in the article/Supplementary Material, further inquiries can be directed to the corresponding author.
